# Supplementary material for: Are carbon emissions trading and green financial instruments synergistic? -Comprehensive quantitative research based on content analysis
Source: PLoS One. 2024 Mar 7;19(3):e0298601. doi: 10.1371/journal.pone.0298601 (PMC10919601; doi:10.1371/journal.pone.0298601)
Supplement: S1 Data — (ZIP) [file pone.0298601.s001.zip › Data/地级市Z_F工作报告.zip!/╣π╢1⁄2/╔╟═╖╩╨/└┤╫╘╣1⁄2╓┌║┼í╛┬φ┐╦╩2╛▌═°í┐_2013.docx]

各位代表：

　　现在，我代表汕头市人民政府，向大会报告工作，请予审议，并请各位政协委员和其他列席人员提出意见。

　　一、2012年工作回顾

　　2012年，市人民政府在省委、省政府和市委的正确领导下，认真贯彻党的十八大、省第十一次党代会精神和省委、省政府一系列决策部署，以特区扩围为契机，以改革创新为动力，以建设幸福汕头为目标，积极应对国际经济持续低迷、国内经济增速明显放缓的严峻形势，真抓实干，奋力拼搏，全市经济社会呈现良好发展态势。全市生产总值1415亿元，增长9.5%；来源于我市的财政收入220.54亿元，其中公共财政预算收入96.34亿元，增长12.6%。固定资产投资额611.92亿元，增长39.7%，新一轮大投资大建设的热潮初步形成。社会消费品零售总额、港口货物吞吐量、集装箱吞吐量、旅客周转量、金融机构本外币存贷款余额等均实现两位数以上的增长，城市的人流、物流、资金流、信息流进一步汇集，集聚辐射和服务功能进一步增强。一年来，各项工作取得了新的成绩。

　　一是大特区城市规划建设取得新进展。高起点谋划城市发展，编制城市发展战略规划，进一步明确“精致汕头，潮人之都，滨海国际化山水人文都市”城市发展定位，编制成果获国际规划界最高奖。启动新一轮城市总体规划修改，推动城乡一体化发展。“一湾两岸”城市核心圈、“一核多组团”的大特区城市新格局初具规模，东海岸新城形成陆域面积超过12平方公里，项目累计完成投资额44亿元；粤东物流产业园项目不断推进，投资8000多万元的展示中心投入使用，设立“汕头市龙湖区电子商务产业基地”和“共建粤东现代物流发展示范区”；濠江新城与中信地产正式签订《城市运营协议》等8个专项协议，南滨片区开发建设项目立项前期准备工作加快推进，苏埃通道项目可行性研究报告获省发改委批准，启动设计招投标；西部智慧生态新城先期启动现代物流商务新城等项目建设；珠港新城总部园区建设正式拉开序幕，出让三块总部企业用地并与中标单位签订发展协议。城市更新逐步推进，汕樟北路、黄河路、金湖路、长平路、外马路、滨港路等一批破旧道路改造完成，建成绿道82公里，城市面貌有所改观。

　　二是重大交通基础设施建设实现新突破。汕揭梅高速公路月浦路段建成通车，结束了汕头近10年没有新开通高速公路的历史，泰山路段、外砂路段加快推进。起点于濠江，经潮阳、潮南、普宁至揭西，全长43.5公里的汕湛高速公路汕头段，起点于潮州，经揭阳、潮阳至惠东，全长16.3公里的潮惠高速公路汕头段启动建设，潮汕二环高速公路纳入省高速公路网规划，工程可行性研究通过省交通运输厅审查，高速公路对内对外互联成网的格局逐步形成。干线公路升级改造进展顺利，南澳大桥全面加快建设，国道324线澄海段、省道233线潮汕路、省道237线和惠公路改造工程（一期）、陈沙公路西延工程建成通车，国道206线大学路等一批公路改造项目相继开工建设。经过多方不懈努力，厦深铁路进汕头站联络线工程可行性研究报告获铁道部和省政府联合批复；厦深铁路汕头段建设顺利推进，潮阳站扩大建设规模方案经铁道部批复同意，已开工建设，潮阳站周边路网及配套设施建设项目已签订合作协议；阜鹰汕铁路汕头至梅州段、汕头港疏港铁路前期工作积极推进，对外联接的铁路通道进一步完善。海门华能煤炭中转基地15万吨级码头和10万吨级深水航道已建成，预计年新增吞吐能力2200万吨。广澳港区防波堤工程加快推进，5万吨级航道工程进入竣工验收阶段。

　　三是产业转型升级呈现良好态势。大力实施“四大产业计划”及相关配套政策，三次产业占比为5.8∶51.5∶42.7，现代服务业和先进制造业投资分别占全市固定资产投资的36.9%和10.7%，产业结构日趋优化。重点项目集中开工季活动成效显著，集中开工项目166个，总投资额336亿元，其中生产性项目投资额173.5亿元，占比超过50%。中国锆城与省经信委共建战略性新兴产业基地，总投资超过23亿元的首期项目全面开工建设；粤东物流新城与阿里巴巴、苏宁电器等签订投资意向项目31个，总投资额约90亿元；汕头卫星应用产业园区、西北航空科技飞机刹车片、猛狮纯电动汽车研发等一批战略性新兴产业项目，苏宁电器、百脑汇等七大商业综合体建设加快推进，工艺玩具、纺织服装、建筑业等传统优势产业保持良好发展态势，现代效益农业稳步发展。澄海玩具基地被认定为第二批国家外贸转型升级专业型示范基地，潮南区获得“中国内衣家居服装名城”称号。国家电子商务示范城市创建工作有序开展，制定出台促进电子商务健康快速发展的若干意见等政策措施，四大电子商务产业园区建设顺利推进，4个项目纳入国家电子商务应用试点，全市电子商务交易规模突破200亿元。电信服务业和互联网服务业的装备水平和通信能力居全国同类城市前列，入选第三届中国城市信息化50强。制定实施加快总部经济发展的若干意见及配套文件，组织认定了首批39家汕头市总部企业，总部经济初具规模，实现产值362.47亿元、税收19.7亿元。鼓励扶持企业开展技术创新，新增专利授权6583件，增长50.6%，其中发明专利184件，增长23.5%；参与国家、行业标准和省地方标准44项，制定产业联盟标准22项，承担省标准化技术委员会3个，国家玩具质量监督检验中心（汕头）获得3C认证出厂检验资质。鼓励扶持企业上市，新增上市企业2家，全市上市企业增至21家，IPO在审企业8家。全年新登记各类企业3224户，其中注册资本超过1000万元的116户，产业经济发展势头向好。节能减排完成年度目标任务。

　　四是开放合作水平不断提升。加快实施“走出去”战略，搭建企业“走出去”平台，举办汕头─洛杉矶、汕头─温哥华经贸合作恳谈会等投资洽谈活动，加快企业境外投资合作步伐。全市现有境外投资企业106家，中方投资总额3.71亿美元。出台一系列鼓励进出口的便利措施，大力推进电子口岸建设，大通关环境进一步优化。鼓励本土潮商走向全国，全国各地设立潮汕（汕头）商会超过60家，成为支持汕头经济发展的重要力量。实施乡贤反哺工程，引导国内外潮商积极参与家乡经济和公益事业建设，成立省潮商公益基金会、潮阳公益基金会和潮南公益基金会，共募集资金超过16亿元。与加拿大潮商集团、深圳潮汕商会、深圳汕头商会等签订战略合作框架协议，投资建设华侨养老院、厦深铁路潮阳站配套设施、“潮人创业园”等项目。积极推进汕潮揭同城化和粤东一体化发展，实现车辆年票通行费互通互认。积极参与海峡西岸经济区建设，依托粤台物流园区、南山湾科技园、台湾农民创业园、南澳论坛等载体，不断深化对台经贸文化交流合作。

　　五是民生民安逐步改善。把保障和改善民生作为公共财政优先方向，优化支出结构，去年全市财政用于民生支出125亿元，占公共财政预算支出比重72.1%，比2011年提高4个百分点。潮阳区、潮南区列入全国县级基本财力保障范围，全市区县建立基本财力保障机制，基本公共服务均等化加快推进。深入开展“三打两建”专项行动，“三打”综合考核居全省前列，社会信用体系建设列入省试点。加强对治安重点地区和突出问题的排查整治，推进社会治安立体防控工作，全市刑事和治安警情下降24.5%，“两抢”警情下降29.2%，下降幅度居全省第一，社会治安形势明显好转。加强出租车综合整治，群众反映强烈的不压表、拒载、乱收费等问题得到有效整顿。深入开展“安全生产年”活动，强化“一岗双责”，落实企业安全生产主体责任，未发生重大以上生产安全事故。提前完成扶贫开发“双到”工作任务，脱贫率达100%。全市就业形势总体稳定，新增城镇就业8.33万人，农村劳动力转移就业4.67万人，城镇登记失业率为2.4%。社会保障覆盖面进一步扩大，全市参加企业职工医疗保险、养老保险47.16万人和80.74万人，城乡居民医疗保险、养老保险444万人和178.9万人。卫生、教育硬件设施不断完善，市中心医院新门诊大楼、汕大医学院附二院医疗中心大楼投入使用，启动建设3家区级人民医院、5家镇卫生院、4家社区卫生服务中心，新增床位470张；新、改、扩建校舍约300所，中小学校舍安全工程竣工率达100%，新增学位约1万个。人民生活水平逐步提高，城镇居民人均可支配收入20024元，农村居民人均纯收入9032元，分别增长14.6%和14.4%。普惠型殡葬惠民政策有序推进，覆盖120万人口。第五次荣获“全国双拥模范城”称号。

　　去年确定的十大民生实事按时完成年度任务：实施千村环境卫生整治，开展垃圾专项治理，完成垃圾场站点规划布点，规划新建垃圾转运站82个、垃圾收集点约4000个，村容村貌有所改善；向373所贫困山区、老区农村小学在编教师每人每月发放300元生活补贴；新投放171台公交车，全市每万人公共汽车拥有量在全省排名第7位，中心城区一票制公交线路、公交轮渡“一卡通”投入使用，调整和增设中心城区道路停车泊位1465个；新建平价商店88家，累计已达137家，发放临时价格补贴762万元；制发加载金融功能社会保障卡160.5万张，对全市60周岁以上未定期领取退休养老金的居民，按每人每月55元发放基础养老金；中心城区所有公园实现免费开放；管道燃气建设投资3471万元，新增管道燃气用户8359户；启动村村通自来水工程，潮阳、潮南区列入省第一批村村通自来水工程示范县，获省2.6亿元资金扶持；建成11个文化站、338个文化室，免费开放中泰运动场，开工建设汕头市全民健身广场；建设保障房4754套，发放租赁补贴1200户，改造农村低收入困难群众危房1350户。

　　六是重点领域改革成效明显。注重运用特区立法，向市人大常委会提请审议法规议案3件，制定政府规章10件。在全省率先出台社会组织登记管理办法，社会组织从1373个增长至3579个，超额完成“十二五”规划发展目标；率先出台电子商务促进办法，推动电子商务健康有序发展；出台医疗保障规定、公租房保障办法、土地储备办法，实施中心城区国有建设用地预申请制度，引领重点经济、民生领域改革。市政府与国家民政部、省民政厅签订民政工作改革创新综合观察点共建协议，成为全国唯一的建立部厅市直通互动工作机制的地级市，为探索民政工作体制机制创新提供强大动力。完成濠江区行政体制综合改革试点工作，出台《行政体制综合改革意见》，全面铺开县级大部门体制改革。创新金融改革，新入驻银行、证券、保险等金融机构11家，第一家股权投资基金注册成立，小额贷款公司、融资担保机构稳健发展，城信社全面退出市场，荣获广东省“金融稳定奖”，金融发展环境进一步改善。出台市级财政专项资金竞争性分配绩效管理暂行办法，开展战略性新兴产业、现代服务业、科技经费专项资金竞争性分配改革试点，重点扶持高科技创新型企业发展。

　　七是政府建设明显提质提效。认真执行市人大及其常委会决议，自觉接受人大的法律监督、工作监督和政协的民主监督。办结人大代表议案（建议）84件，政协提案177件。出台法治政府建设五年规划，组建法律咨询专家委员会，促进行政决策科学化、民主化、法治化。在北京大学成功举办行政程序研修班暨高层论坛，着力提升全市领导干部依法行政的素质和能力。继续深化行政审批制度改革，取消、转移、下放、前移政府职能事项达53%。推进行政审批流程再造，压缩企业投资和工程建设项目并联审批时限，加大重大项目全程无偿代办服务力度。完善重点项目建设责任制和倒逼机制，全面推行重点项目承诺制。加快电子政务建设，全面启动“汕头政府在线”项目，42个单位358项行政审批和便民服务事项梳理上网，“网上办事大厅”成功并入省网，成为全省首批上线城市，荣获国家“2012年度电子政府创新应用奖”。市网络问政平台上线运行，打造群众问政参政议政机制平台。深入开展“治贪、治庸、治懒、治散”专项整治行动，加强行政监察和审计监督，政府作风、行政效能、政务环境明显改善。

　　各位代表！过去一年，我们所取得的成绩和进步，是省委、省政府和市委坚强领导的结果，是全市人民同心协力、共同奋斗的结果。在此，我代表市人民政府，向奋斗在各条战线的全市广大干部群众、外地来汕建设者，向给予我们支持的人大代表、政协委员、各民主党派、工商联、社会各界人士和海内外乡亲，向中央和省驻汕单位，向驻汕解放军、武警官兵，向所有关心和支持汕头发展的港澳台同胞、海外侨胞和国际友人表示崇高的敬意和衷心的感谢！

　　我们取得的成绩来之不易，但与中央和省的要求相比，与人民群众、海内外潮人的期盼相比，我们的工作还存在很大的差距和不足，经济社会发展还有很多亟待解决的问题：一是经济持续较快增长的动力和后劲不足，产业结构亟待优化，加快发展和转型升级的双重任务很重；二是政府财力薄弱，教育、医疗、住房、社会保障等民生事业欠账多，与人民群众的需求差距仍然很大，影响社会和谐稳定的因素还很多；三是社会管理不到位，交通秩序、市容市貌、生态环境还没有明显改观，社会治安压力依然很大，群众反映强烈；四是阳光、法治、服务政府建设任重道远，有些公务员服务观念淡薄，政府服务水平、行政效能需进一步提升。我们必须正视自身工作中存在的问题，采取有效措施认真解决。

　　二、2013年工作安排

　　今年是全面贯彻落实十八大精神的开局之年，也是实施“十二五”规划承前启后的关键一年。我们将认真落实党的十八大、中央经济工作会议、省委十一届二次全会、市委十届三次全会精神，以科学发展为主题，以建设美丽幸福汕头为目标，坚持根植性发展、精致型开发、包容性增长理念，深化改革开放，全面统筹城乡发展，加快推进产业转型升级，加强社会管理创新，切实保障和改善民生，推动经济社会发展迈上新台阶。

　　今年经济社会发展的主要预期目标是：地区生产总值增长10%，公共财政预算收入增长12%，固定资产投资额增长25%，居民消费价格指数涨幅控制在3.5%左右，城镇居民人均可支配收入增长10.5%，农村居民人均纯收入增长11%，城镇登记失业率控制在3%以内，节能减排完成年度目标任务。重点抓好六方面工作：

　　 （一）着力打好交通基础设施建设大会战

　　新的一年，要继续集中资源、集中力量，积极争取上级支持，千方百计攻克征地拆迁、资金、项目审批等难题，打响打好交通基础设施建设大会战。

　　高速公路方面。加快汕揭梅高速公路汕头段建设，月浦至泰山立交段实现通车。加快推进汕湛高速公路、潮惠高速公路汕头段建设，力争起点于揭阳，经潮阳、潮南至惠来，全长38公里的揭惠高速公路汕头段尽快开工建设。争取潮汕二环高速公路获批立项。

　　铁路方面。全力配合厦深铁路汕头段建设，确保按省部要求按时通车。深化与战略投资者的合作，加快推进厦深铁路潮阳站进出站路建设，确保与厦深铁路同期通车。力争厦深铁路进汕头联络线工程开工建设，继续推进汕头港疏港铁路、阜鹰汕铁路梅州至汕头段前期工作。积极配合省组织编制粤东城际轨道交通规划，完成汕头市域轨道交通线网规划编制。

　　路桥建设方面。建成南澳大桥，破解长期制约南澳发展的交通瓶颈。完成国道324线外砂段、国道206线大学路一期改造工程，启动实施国道206线大学路二期、国道324线潮阳梅花至潮南洋汾陈段，省道231线澄海路段、省道234线揭海公路北闸至金灶段、省道235线司神公路两英至雷岭段、省道237线和惠公路二期及河浦大道、磊广路、河中路、达南路等改造工程建设，加快提升路网通行服务水平。继续完善危桥整治，加快农村公路建设。加快推进枢纽站场建设，积极配合省机场集团建设城市候机楼。

　　港口方面。大力推进广澳港区防波堤工程建设，确保工程按计划顺利推进。开工建设广澳港区10万吨级集装箱泊位、2万吨级石化码头，改造马山港区华能汕头电厂重件码头。全面建成粤东煤炭中转基地并投入正式营运。启动广澳港区航道二期工程前期工作。

　　（二）着力提高产业整体素质

　　实现有品质、高效益、可持续的发展，关键是推进产业集聚和转型升级。要始终加强产业规划引导和政策扶持，在充分发挥侨乡、特区优势，策划、包装、推介一批重大产业合作项目，更加注重结合本地产业需求的产业链招商和先进制造业招商，推动现有签约重大项目的落实，扶持本土企业做强做大、转型升级，构建品质效益型精益经济。

　　谋划推进一批产业项目。继续推进中国锆城、“数据特区”、卫星应用产业园区、西电全数字型智能电源电站扩建等一批重大战略性新兴产业项目，苏宁电器、长江路北、星湖城、百脑汇、红星美凯龙、华润万家、广厦正大广场等一批商业综合体项目，粤东物流新城、澄海国际玩具商贸物流城、广澳国际集装箱物流中心、谷饶国际针织内衣商贸城、潮南纺织产业创新基地等一批物流项目，以及潮博园等一批潮汕文化展示平台建设。加快“汕头生态旅游示范区”建设，重点打造南澳岛旅游终极目的地，启动潮人码头文化公园建设，规划国际邮轮码头、游艇基地、帆板基地等一批高端滨海旅游项目。推进农业标准化、规模化、产业化、社会化，大力培育一批现代农业园区、农业龙头企业、观光农业基地。加快创建国家电子商务示范城市，推动苏宁易购等一批电子商务平台项目、电子商务园区建设。按照“四大产业计划”，结合各产业园区功能定位，高水平策划包装，面向央企、民企和海内外潮商招商，重点引进一批附加值高、产业链长、带动力强的重大项目，在第五届粤东侨博会期间开工、签约一批项目。

　　破解产业发展瓶颈。继续完善各种扶持产业发展配套政策措施，推动产业转型升级。支持鼓励金融改革创新，加快发展村镇银行、创业（风险）投资、小额贷款、融资担保等各类机构，研究探索利用公司债、企业债、集合债等多种债务融资工具，进一步拓宽企业直接融资渠道，促进金融与实体经济的良性互动发展，解决产业发展融资难问题。大力推动企业改制上市，争取新增上市企业3家以上。努力推动农村金融工作，启动汕特农信联社改革，继续推进潮阳、澄海、南澳三家农信社改制，争取澄海农信社完成农村商业银行改制，积极服务“三农”发展。加快推进“三旧改造”，开展城乡建设用地增减挂钩及低丘缓坡荒滩等未利用地开发利用试点。全面实施用地指标竞争性分配机制，对开工建设条件成熟、符合产业发展导向的项目实行优先供地，满足产业发展用地需求。助推大型骨干企业和中小微企业加快发展，启动100家重点企业做大工程、100家战略性新兴产业企业培植工程、100家乡贤反哺企业落地工程、100家传统优势产业企业转型升级工程、100家现代服务业企业培强工程，培育、扶持一批具有核心竞争力、高增长性、经营规模超10亿、100亿的大型骨干企业尤其是民营企业。

　　推动产业集聚发展。全力加快省产业转移园各片区基础设施建设，抓好园区二次开发，整合现有工业园区，集约使用有限建设用地建设民营工业园、通用厂房，引导企业进园区，增资扩产、集聚发展。重点招引高端制造业和高新技术产业项目进园发展。研究出台鼓励企业进园区的政策措施，完善市内各区产业转移的利益分配机制，形成布局合理、特点鲜明、配套完善、集约发展的产业发展新模式。充分发挥高新区在人才聚集、科技服务、信息服务上的优势，重点扶持孵化一批科技创新型项目和企业扩大规模，发挥效益。加速推进保税区转型升级为综合保税区，发挥政策功能优势，以新办高端企业和大宗商品交易市场为载体，实现内外联动、区港联动，促进区港一体化发展。会同口岸联检部门进一步实施通关便利措施，加大力度优化大通关环境，保持进出口稳定增长，培育提升汕头港作为区域性大宗物资和原材料进口港的地位。

　　支持企业自主创新。实施创新驱动发展战略，优化产学研合作环境，促进企业与大型央企、高等院校、科研机构联合研发和引进科研成果并实现产业化，鼓励建立技术联盟、产业联盟和标准联盟，推进传统优势产业转型升级。建立健全科技经费正常增长机制，强化财政资金向拥有自主知识产权和有市场竞争力的项目倾斜，切实发挥财政资金的引导作用，促进经济发展质量提升。进一步扶持产业共性技术研发和产业科技创新平台建设，加快建设国家日用精细化工产品质量监督检验中心、国家玩具检测重点实验室、国家纺织品安全检测重点实验室、轻工装备研究院等一批公共技术服务平台，促进企业自主创新能力的提升。围绕产业发展需求，大力引进、培养一批创新型、高层次人才，以及高技能实用型人才。

　　（三）着力推进城市扩容提质

　　加快推进城镇化、实现城乡一体化发展是大特区城市发展的必由之路。必须以城市扩容提质为抓手，以生态带型都市和精致空间的梳理打造为核心，统筹功能与设施布局，打造城乡经济社会发展的优越空间载体和要素。

　　加快推进海湾新区建设。高标准、高起点完成汕头海湾新区总体规划编制工作，并做好产业发展、区域建设用海、基础设施建设等3个专项规划。围绕建设华侨经济文化合作试验区，深入开展政策创新研究，在推动华侨经济与文化发展、华侨华人资产管理、对台合作、金融创新、用地用海等方面，争取国家给予先行先试政策支持，打造全国华侨经济文化合作发展的重大平台。加快推进海湾新区各功能组团建设，尽快实施新东区规划，东海岸新城基本完成围内土地吹填和海堤、闸站及内河涌建设，启动市政干道及连接桥梁建设；濠江新城项目完成南滨片区12平方公里土地平整，力争上半年启动中轴线景观工程、中信滨海新城体验馆等基础设施建设，年底前启动苏埃通道建设；珠港新城完成控制性详细规划编制，整合盘活土地资源，启动总部经济园区建设；粤东物流新城加快启动建设。

　　推动城乡综合运营开发。创新城乡发展投融资机制，加强城市建设开发总公司、投资总公司等城乡发展投融资平台建设，充分利用各种金融杠杆和市场融资功能，努力破解城乡建设、城市更新的资金瓶颈。推进城市土地储备经营，盘活低效用地，做大土地储备总量，重点推进珠港新城、金凤半岛、汕大南侧、莲塘片区、新溪片区等储备项目。开放市政公用事业、城乡基础设施建设市场和运营市场，积极引进战略投资者，实施综合运营开发。启动西部智慧生态新城土地一级开发和金凤西路、金环西路、金砂西路等路网基础设施建设。加快潮阳东部新城、潮南行政新区、厦深高铁潮阳站（谷饶）物流新城等一批项目的战略合作开发。加强监管和搞活并举，加快国企改革步伐，建立国有资本经营预算制度，整合盘活国有资产和资源。继续发行城投债和企业债券，最大限度发挥资金杠杆作用，撬动和推进一批带有全局性、利于滚动开发的重大基础设施和产业项目建设。

　　加强城市综合管理。启动中山中路、长平西路、金砂西路、外马路等一批老城区破损道路、“水浸街”更新改造，以实施永平路保护性开发为示范，推动小公园历史文化街区的保育活化，打造全国开埠商业文化活体博物馆群，进一步改变西片区城市面貌。推进西堤路和泰山路连接高速公路配套工程，改善城市主出入口形象。加快市区道路LED路灯改造，推进重要路段和区域城市景观照明设施的建设及维护。加快城市管理体制机制创新，构建网格化、数字化管理模式，推动环卫、园林绿化、市政设施维护等作业层面市场化。深入开展综合整治行动，启动小贩疏导工程、集贸市场升级改造工程，大力整治城市管理“顽疾”。加快推进城市交通智能化，全面完成二期智能交通管理系统建设，推进新建、扩建道路与智能化交通管理系统同步规划、同步实施、同步建设。推行居民小区、学校、医院、商业中心周边区域道路交通“微循环”管理以及背街小巷错时停车便民服务，科学细化交通信号灯配时，进一步优化城区交通流组织，缓解中心城区交通拥堵现状。持之以恒开展交通秩序综合整治，严肃查处各种交通违法行为，坚决取缔无牌无证、假牌假证机动车辆，严格出租车营运管理。坚持公交优先发展战略，重点调整优化线路布局，提升城市公交服务水平，为广大人民群众提供便捷、安全、舒适的公交服务。

　　着力改善城乡生态环境。把生态文明建设放在突出位置，重点解决影响人民群众生活质量的生态环境问题。启动国家生态园林城市、国家森林城市创建工作，动员全社会力量组织实施“绿满家园”行动，通过推进景观林带建设、道路绿化改造、城乡见缝插绿、增加园林小品、桥梁垂直绿化，打造出门见绿、四季花开、满城皆景的富有潮汕特色的城市绿化景观。继续开展“千村环境卫生整治”，从根本上解决农村“垃圾围村”问题。加强城乡环境保护和监控执法力度，积极推进环保电厂、污水处理厂及配套管网建设，提高城镇生活垃圾无害化处理率及污水收集率。继续强力推进练江流域污染综合整治及重污染行业整治工作。组织开展宜居城镇、宜居社区、宜居村庄评选和创建活动，推荐一批社区申报“省级宜居示范社区”。大力推进农田水利基础设施和防灾减灾工程建设，完成汕头大围等一批重点水利工程。支持南澳创建“国家级海洋生态文明示范区”，发展海洋清洁能源，推进海洋生态保护与修复。

　　推进汕潮揭同城化。进一步完善同城化工作机制，全面实施公交车出租车辆相关资格互认、广播电视互相落地播出、通信同网同费、医保联网结算收费、医疗检查结果和病历互认等同城化合作项目，推动汕潮揭同城化向纵深方向和更高层次发展。

　　（四）着力创新社会管理和公共服务

　　积极回应群众的期待和诉求，把群众对美好生活的向往，作为我们工作的目标，大力创新社会管理和公共服务，努力为群众提供更好的教育、更稳定的工作、更满意的收入、更可靠的社会保障、更高水平的医疗卫生服务、更舒适的居住条件。

　　推动民政工作改革创新。加快民政工作改革创新综合观察点建设，创新基层社会管理服务体制，探索建立城市社区公共服务站，开展“一站多居”或“一站一居”治理模式试点，理顺社区居委会与基层政府及社区其他组织的关系。推进社区居委会小型化试点，探索居委会成员属地化、兼职化。深化社会组织管理体制改革，重点培育发展行业协会商会、枢纽型社会组织和公益慈善类社会组织。开展公益慈善组织社会化运作改革，打造一批具有潮汕特色的慈善组织品牌。编制社会组织承接政府职能和获取政府购买公共服务名录，拓展社会组织发展空间，促进社会组织健康快速发展。积极探索适合本地的社工发展模式，培育发展民办社工服务机构，开发一批社会工作岗位，探索“社工+义工”联动机制。全面推进居家养老服务示范活动，建设第二批城市居家养老服务示范中心。扎实推进普惠型殡葬惠民政策实施。巩固“全国双拥模范城”创建成果，提高双拥共建工作水平。

　　大力促进教育发展。加快教育创强工作，均衡发展九年义务教育，合理配置教育资源，重点向农村、边远、贫困地区和特殊群体倾斜，全市超过80%的义务教育学校达到规范化学校建设标准。积极推动农民工子女平等接受教育，支持特殊教育，尽快建成1所市级、3所区级特殊教育学校，让每个孩子都能成为有用之才。办好学前教育，确保85%农村乡镇建成普惠性规范化中心幼儿园。全面提高普及高中阶段教育的整体水平，突出发展中等职业技术教育，推进市鮀滨职校、金平区职教中心、澄海区职教中心、潮南区职教中心、林百欣科技中专等中职学校校舍建设，全市高中阶段教育毛入学率保持在90%以上。支持汕头大学建设成为国内一流大学，加快把汕头职业技术学院建设成为省级示范性高职院校。加强教师队伍建设，严格落实义务教育学校绩效工资制度，确保中小学教师工资福利待遇“两相当”。

　　切实改善医疗卫生公共服务。完善基层医疗卫生机构财政投入机制，加大基层医疗机构改造力度，加快建设3家区级人民医院、4家社区卫生服务中心和5家镇卫生院。启动汕头大学医学院附属肿瘤医院重建工作，加快推进市第二人民医院改扩建，策划在东区、西区各建2家医院。实施便民就医工程，继续推进城市对口支援基层医疗服务计划，为边远地区提供巡回医疗服务等多种支持，逐步形成基层首诊、分级医疗、双向转诊的服务模式。鼓励高等院校医学类毕业生到基层医疗卫生机构工作，提高基层医疗卫生服务水平。深化医药卫生体制改革，全面实施国家基本药物制度，积极推进公立医院改革试点。落实2个国家级全科医生规范化培养基地建设，加快推进市卫生学校易址重建。加强人口计生服务和管理，稳定低生育水平，促进人口长期均衡发展，提高人口素质。

　　完善多层次社会保障。实施就业优先战略和更加积极的就业政策，新增城镇就业6万人，农村劳动力转移就业4万人。加快城乡一体化社会保障体系建设，强化巩固社会保险扩面征缴工作，实现全市城乡居民养老保险全覆盖，力争完成社会保障卡发放，加快推进“金保工程”（二期）建设，开展城乡居民医疗保险引入市场机制试点。加快推进残疾人社会保障体系和服务体系建设，加大对孤儿、残障儿童、贫困精神病患者等特殊群体救助力度。

　　加快文化体育事业发展。深入发掘特区文化、华侨文化、海洋文化和潮汕传统文化的丰富内涵，大力弘扬开放兼容的人文精神。大力实施文化惠民工程，推进基层文化站、文化室、科普教育基地等设施建设，广泛开展群众性文化活动，提高全民文明素质。做好文化遗产保护，继续支持红色文物修缮工作，支持侨批档案申报联合国记忆遗产名录。广泛开展全民健身活动，完善城乡社区体育运动设施，不断完善全民健身站点。

　　深入推进平安创建工作。全面开展平安区县、平安乡镇、平安社区等基础创建活动，推进群防群治工作，加快社会治安视频监控和治安防控设施建设，着力打造人防、物防、技防、制度防“四位一体”的社会治安立体防控体系。

　　（五）着力办好十大民生实事

　　今年市级财政投入6.7亿元，办好十大民生实事。

　　1、实施“绿满家园”全民行动。围绕“打造绿色生态城市，建设美丽幸福汕头”主题，启动一批城市公园、一批森林生态项目建设改造，实施城乡道路、单位大院、社区、庭院绿化美化。建成海湾湿地生态公园并在“五一”前免费开放。

　　2、继续推进千村环境卫生整治。重点推进垃圾专项整治，建设一批垃圾填埋场、转运站、收集点等环境卫生基础设施，实现农村垃圾“村收集、镇转运、区县处理”。在全市范围开展灭鼠、灭蟑集中行动。

　　3、继续推进城乡保障性住房建设。筹集公租房1300套，发放租补1200户，完成4072户农村低收入住房困难户住房改造任务。调剂限价房、公租房各100套，优先解决乌桥片区住房困难户、危房户的居住问题。同时，按每户1.5万元标准，资助建设海门渔民安居房1005套。

　　4、实施潮阳、潮南区村村通自来水工程。用两年时间（2013-2014年）实施潮阳区、潮南区村村通自来水工程，实现两区行政村自来水覆盖率90%以上、农村自来水普及率90%以上、农村饮用水水质合格率90%以上的工作目标。

　　5、提高义务教育生均公用经费标准。小学每人每学年从550元提高到750元，初中每人每学年从750元提高到1150元。

　　6、实施全民健康工程。提高城乡居民基本医疗保险补助标准，每人每年从240元提高到280元，实现全市医保覆盖率达到95%以上的目标；提高对居民健康档案、健康教育、儿童保健、孕产妇保健等11类基本公共卫生服务的补助标准；全面推行免费孕前优生健康检查，提高出生人口素质；选择一批医疗机构实施“平价医院”、“平价诊室”、“平价药包”服务，降低群众看病、购药费用；进一步铺开低成本健康工程，方便人民群众就诊就医。

　　7、提高养老保障服务水平。巩固和提高城乡居民社会养老保险参保率；建设一批社会养老服务设施，启动建设市社会福利中心，建设一批老年人日间照料服务中心、农村养老服务示范点和老年人活动场所，资助民办养老服务机构开展养老服务。

　　8、提高城乡最低生活保障补贴水平。各区县低保补贴水平达到城镇平均每人每月242元、农村平均每人每月109元。

　　9、加大特殊群体救助力度。资助200名先天性心脏病儿童接受手术治疗；资助需要帮助的30至50岁的贫困家庭风湿性心脏病患者接受手术治疗；为1000名贫困重性精神病患者提供住院医疗救助；资助4000名贫困精神病患者免费接受社区服药诊治；资助500名贫困白内障患者免费接受复明手术；资助400名学龄前残疾儿童接受抢救性康复救助；为9000名极重度（一级）残疾人和8000名重度（二级）残疾人提供护理补贴。

　　10、推进文化城乡全覆盖。市博物馆新馆春节期间正式开馆并免费开放；达标升级建设10个综合文化站、300个行政村（社区）文化室；在全市541个行政村实施农村公益电影放映工程，每村每月放映一场公益电影，全年放映农村公益电影6492场；组织潮剧潮艺演出团体进社区、进农村、进企业演出。

　　（六）着力提升政府行政效能

　　提高政府行政效能，必须创新行政管理方式，深化行政审批制度改革，坚持不懈推动阳光法治服务政府建设。

　　坚持科学民主依法决策。必须自觉接受市人大及其常委会的法律监督、工作监督和政协的民主监督，主动听取各民主党派、人民团体和社会各界的意见建议，问需于民，问计于民，问政于民。坚持依法行政，加强和改进政府立法，完善法规规章，最大限度减少体制和制度漏洞。按照《行政程序规定》，创新决策机制，充分发挥法律咨询专家委员会的智囊效应，加强发展战略和重大项目决策合法性论证，严格规范决策程序。

　　深入开展行政审批制度改革。建立健全审批事项动态清理公示机制，优化再造审批流程，推进行政审批三集中制度，促进行政审批提速增效，实现投资领域办理时限缩减50%以上。组织实施“汕头政府在线”项目，抓紧建设各区县网上办事大厅，推进市、县（区）、镇（街）三级联网。开展市直单位办公自动化系统试点工作，促进“汕头政府在线”项目普及应用。出台实施《汕头政府在线管理办法》，提高网上办事大厅服务集成化水平，健全市行政服务中心建设，促进网上网下行政服务有效衔接，更好地为社会公众提供一站式服务。全面推行网上审批，加强网上电子监察系统建设，完善审批事项实时在线监督。继续推动网络问政，促进民意征集常态化。

　　大力推进大部门制改革。探索市级大部门制改革，理顺大部门职能关系，完善部门职责分工协调机制，开展相对集中行政处罚权改革，着力解决推诿扯皮、权责脱节问题。推动政府职责关系法定化，加快富县强镇和简政强镇事权改革，扩大下级政府管理权限，微观事权和执行权下移，促进政府扁平化管理。积极稳妥推进事业单位分类改革。

　　积极推进社会信用体系、市场监管体系建设。加快推进社会信用体系试点工作，重点在政务诚信、信用立法、公共联合征信和统筹城乡金融服务、食品药品、教育、医疗卫生等领域取得实效，优化营商环境。推进市场监管体系建设，建立全市统一的市场监管信息平台，重点推进市场准入、质量监管、市场交易监管、消费维权、食品安全等五大信息系统。严格落实安全生产“一岗双责”制度，加强安全生产监督管理，突出重点行业领域事故隐患排查治理，遏制较大以上安全生产事故，努力实现安全生产形势根本好转。持续不断开展经济户口清理，依法规范所有工商户生产经营证照，加快推进“综合治税涉税信息交换和共享系统”建设，推行“阳光税务”，落实税负公平，引导企业自觉承担社会义务和社会责任，共同营造可持续发展环境。

　　切实改进政府作风。严格执行中央关于改进工作作风、密切联系群众的八项规定，坚决克服形式主义、官僚主义，下决心改进文风会风，精简会议活动和文件简报。改进领导干部公务活动安排，切实做到轻车简从、简化接待、减少陪同。坚持艰苦奋斗、勤俭节约，加强经济责任审计，大力整治贪庸懒散等不良作风，推动形成为民、务实、清廉的政风。

　　各位代表，今天的汕头已经站在一个新的发展起点上，美好前景激励着我们，人民群众期待着我们。让我们在省委、省政府和市委的坚强领导下，团结带领全市人民，凝聚海内外潮人的智慧和力量，珍惜当前来之不易的良好发展势头，乘势而上，勤勉工作，努力向全市人民交出一份合格的答卷！
